# New opportunities rising

**DOI:** 10.7554/eLife.26775

**Published:** 2017-05-09

**Authors:** Jessica C Thompson

**Affiliations:** Department of Anthropology, Emory University, Atlanta, United Statesjessica.thompson@emory.edu

**Keywords:** Homo naledi, hominin, Dinaledi Chamber, Lesedi Chamber, human evolution, paleoanthropology, None

## Abstract

More fossil specimens and an eagerly awaited age for *Homo naledi* raise new questions and open fresh opportunities for paleoanthropologists.

**Related research article** Dirks PHGM, Roberts EM, Hilbert-Wolf H, Kramers JD, Hawks J, Dosseto A, Duval M, Elliott M, Evans M, Grün R, Hellstrom J, Herries AIR, Joannes-Boyau R, Makhubela TV, Placzek CJ, Robbins J, Spandler C, Wiersma J, Woodhead J, Berger LR. 2017. The age of *Homo naledi* and associated sediments in the Rising Star Cave, South Africa. *eLife* **6**:e24231. doi: 10.7554/eLife.24231

**Related research article** Hawks J, Elliott M, Schmid P, Churchill SE, de Ruiter DJ, Roberts EM, Hilbert-Wolf H, Garvin HM, Williams SA, Delezene LK, Feuerriegel EM, Randolph-Quinney P, Kivell TL, Laird MF, Tawane G, DeSilva JM, Bailey SE, Brophy JK, Meyer MR, Skinner MM, Tocheri MW, VanSickle C, Walker CS, Campbell TL, Kuhn B, Kruger A, Tucker S, Gurtov A, Hlophe N, Hunter R, Morris H, Peixotto B, Ramalepa M, van Rooyen D, Tsikoane M, Boshoff P, Dirks PHGM, Berger LR. 2017. New fossil remains of *Homo naledi* from the Lesedi Chamber, South Africa. *eLife* **6**:e24232. doi: 10.7554/eLife.24232

**Related research article** Berger LR, Hawks J, Dirks PHGM, Elliott M, Roberts EM. 2017. *Homo naledi* and Pleistocene hominin evolution in subequatorial Africa. *eLife* **6**:e24234. doi: 10.7554/eLife.24234

After the discovery of *Homo naledi* was announced in 2015, many mysteries still remained (Stringer, 2015). The fossils were undated, much to everyone’s regret, and there was much debate about where the material should fit in the larger scheme of human evolution. The location of the find in the Rising Star cave system in South Africa also raised an important question: how did so many fossils from a single species arrive so deep in the dark zone of the cave? Now, in three papers in eLife, Lee Berger, Paul Dirks, John Hawks and an international team of colleagues working across seven countries shed light on some of these mysteries, including an estimate for the age of the fossils (Berger et al., 2017; Dirks et al., 2017; Hawks et al., 2017).

From the start, *H. naledi* complicated what otherwise seemed like a straightforward story about human evolution (Berger et al., 2015). The narrative used to be that the first member of the genus *Homo* appeared about 2.5–2 million years ago; that their descendants had spread across the Old World by 1.8 million years ago; and that they evolved notably larger brains and essentially human-like body plans by 1.5 million years ago (Antón et al., 2014). Our own species, *Homo sapiens*, did not appear until much later (between about 300,000 and 200,000 years ago).

With its mix of primitive and derived features *H. naledi* was difficult to place in this simple narrative. The fact that it was undated made it harder still (Hawks and Berger, 2016). Many guessed that it must be closer in time to the origin of *Homo* than to the emergence of *H. sapiens* (Dembo et al., 2016; Francis Thackeray, 2015). However, as demonstrated many times in paleoanthropology, evolutionary processes are complex and we should be wary of overly simplistic scenarios. Now, in the first of the three papers, Dirks et al. deliver a particularly bold dash of complexity, and report that the fossils are most likely between 236,000 and 335,000 years old (Dirks et al., 2017). This date firmly places them within the late Middle Pleistocene, at a time when the earliest members of our own species were making their debut.

Should paleoanthropologists be excited and surprised by this new information? Excited, yes, but perhaps not surprised given that the discovery of *H. naledi* has already been extraordinary in almost every sense. The Rising Star cave system is at the edge of the Cradle of Humankind, a 47,000-hectare World-Heritage-listed site in South Africa that has yielded more hominin fossils than anywhere else on Earth. However, Rising Star had somehow been bypassed by generations of paleoanthropologists until one small excavation three years ago unearthed more than 1500 specimens representing at least 15 individuals (Berger et al., 2015). The find was remarkable to say the least, given that hominin species are often first described from only a few fossils. The *H. naledi* fossils were also found deep inside the cave, unmixed with artifacts or the remains of any other animals (Dirks et al., 2015). Other sites in the Cradle of Humankind, however, contained fossils of many species and were found near accessible entrances; several sites also yielded stone tools. Perhaps most tellingly, and also unlike other Cradle hominins, the Rising Star specimens were largely intact and not heavily fossilized. In short, *H. naledi* was different from the beginning.

Under the original narrative, by the late Middle Pleistocene, all representatives of *Homo* in Africa were quite evolved compared to early *Homo* (Rightmire, 2009). Why then did *H. naledi* seem to have more ancient characteristics, like a small brain, that placed it with early *Homo*, and other characteristics, like the shape of its wrist bones, that seemed to skim past a million years of evolution and be found in later species such as humans and Neanderthals? Now, in a second paper, and in light of the new dates reported in the first paper, Berger et al. put forward a scenario that challenges the original narrative (Berger et al., 2017). They propose that an incomplete and poorly dated record of Middle Pleistocene fossils has created opportunities for other discoveries to be assigned to the wrong species or time period. They argue that this has led to researchers making unwarranted inferences about how many, and what kinds of, hominin species inhabited Africa at any given time.

Although these claims may seem as extraordinary as the findings themselves, the simple narrative across the arc of human evolution has been breaking down for some time. The current situation – with us as a lone, global species – has long been recognized as unusual, compared to much of human evolution in the past. There has also been controversy over how to define our genus *Homo*, and a growing certainty that the earliest archaeological record (e.g. stone tools) was left by many different species (Kimbel and Villmoare, 2016). There is genetic evidence that humans were cosmopolitan breeders, incorporating DNA from "archaic" hominins into our lineage both in Africa and in Eurasia (Hsieh et al., 2016). Instead of thinking about the advantages that one discrete species had over other species, we now need to consider the powerful biological advantages of hybridization (Ackermann et al., 2016).

The new information from Rising Star reinforces growing evidence that different branches of human evolution explored many winding paths across Africa in the Middle Pleistocene (Stringer, 2016). It may even suggest that early members of our own species directly encountered *H. naledi* and other presently unknown hominins. The nature of those encounters in Africa remains to be discussed, as does the role our species may have played in the ultimate extinction of other hominins. All of this is excellent fuel for opening up a new area of research, though the next phase – that of filling in the nuance – is where the hardest work will be.

In a third paper Hawks et al. report the discovery of more fossils of *H. naledi* – including a relatively complete cranium – from the Lesedi chamber in the Rising Star system (Hawks et al., 2017). The first specimens were found in the Dinaledi chamber and the discovery of more specimens from a different cave chamber appears to remove the possibility that the sample from Dinaledi represented a one-time catastrophic event that killed a single group of *H. naledi*. This deepens the lingering mystery of how the fossils came to rest in such dark and inaccessible parts of the cave. Were the dead intentionally placed there after all? The new dates put *H. naledi* in a time and place where there is some of the earliest physical evidence for human culture, including regular controlled use of fire, complex stone tools, and ritualistic behavior with natural pigments (Watts et al., 2016; Wilkins et al., 2012). However, none of this evidence was found with *H. naledi*; it was instead found at other Middle Pleistocene sites in South Africa. Berger et al. suggest controversially that the possibility of a creature like *H. naledi* being responsible cannot currently be discounted (Berger et al., 2017).

Here is a robust challenge that many archaeologists will be happy to undertake, as a century of work in Europe has shown quite clearly that even closely related hominin species do leave different cultural records (Wynn et al., 2016). The Eurasian record further shows that when modern humans encountered such species, they swapped genetic material in the few thousands of years before those other species went extinct. Did this happen to *H. naledi*? Did they themselves leave their dead in that cave, or did something else? Was *H. naledi* capable of making the kinds of stone tools found in the southern African Middle Pleistocene? Did they have a symbolic culture? Although these questions make tempting headlines, it must be emphasized that for every site that has complex stone tools and pigments, there are a thousand more that do not. Well-dated Middle Pleistocene sites are rare, and there are simply not enough datapoints to understand the timing and extent of cultural systems at this time, nor how they could potentially relate to biological populations (Figure 1).

**Figure 1.**
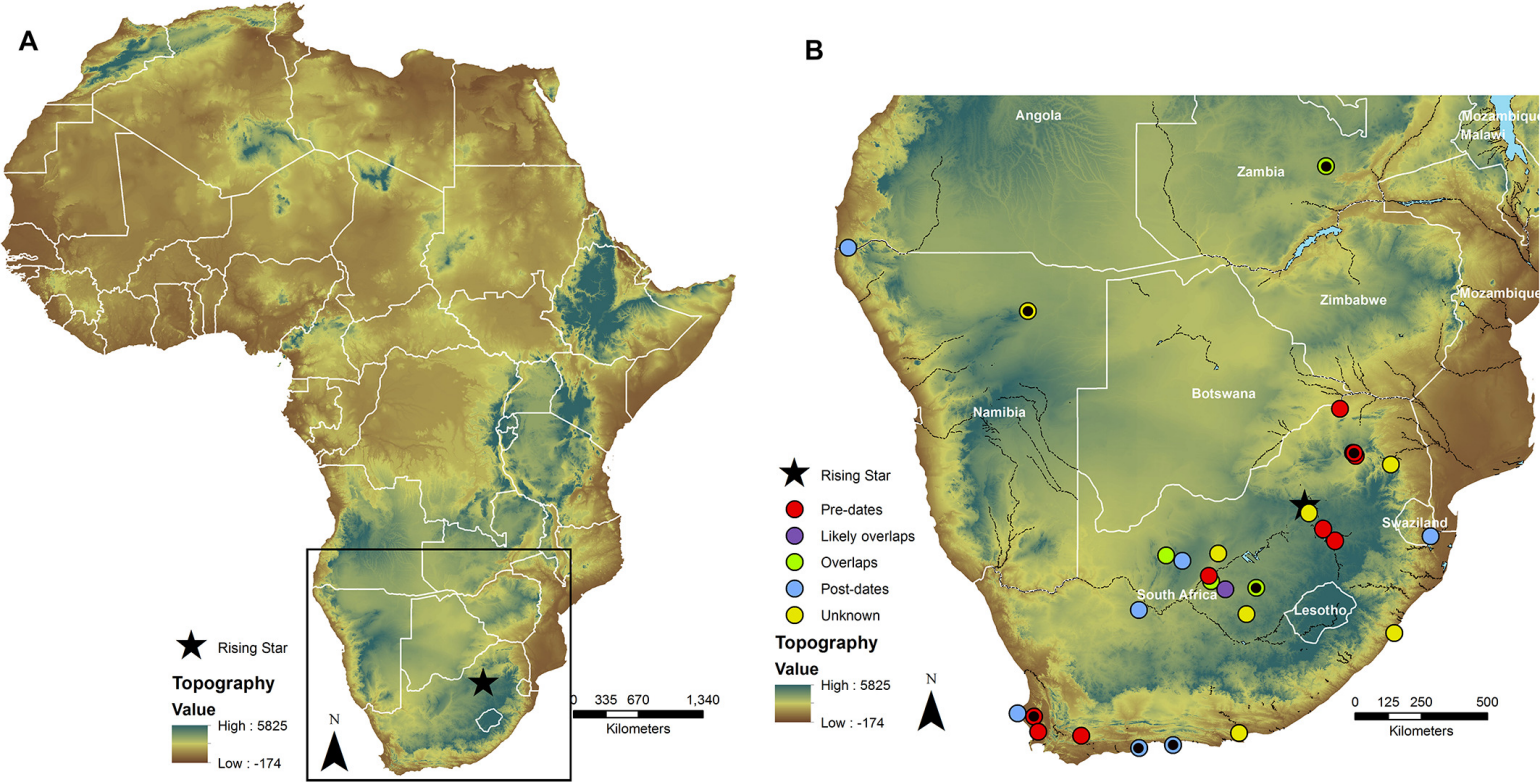
The Rising Star cave system and other archaeological sites in southern Africa from around the Middle Pleistocene. (**A**) The *Homo naledi* fossils were found in the Rising Star cave system (shown with a star). (**B**) A close-up of the boxed area in A shows the distribution of dated Middle Pleistocene or likely Middle Pleistocene archaeological sites in southern Africa. Sites are colored based on their age compared to the Rising Star sites. Sites with hominin remains contain a small black circle. Topography is GTOPO30 data.

The wealth of new data from these three papers is critical for our understanding of the biology, evolution and behavior of *H. naledi*, and its implications ripple across the discipline. However, not all of these ripples are entirely unexpected. Paleoanthropology is experiencing a crossroads in how the human past is – and should be – conceptualized. As a field that is only a few scientific generations old, it continues to mature in the midst of a persistent stream of new discoveries, opportunities and challenges. One of these challenges will be to confront the problem of how to reconcile the biological and archaeological records of a continent where a sparse Middle Pleistocene record has formerly offered few options for doing so. With this opportunity now open, there is much work ahead.
